# Synthesis, spectroscopic analysis and crystal structure of (*N*-{2-[(2-amino­eth­yl)amino]­eth­yl}-4′-methyl-[1,1′-biphenyl]-4-sulfonamidato)tri­carb­on­ylrhenium(I)

**DOI:** 10.1107/S2056989024005656

**Published:** 2024-06-18

**Authors:** Dinithi Kaluthanthiri, Theshini Perera, Frank R. Fronczek

**Affiliations:** a Department of Chemistry, University of Sri Jayewardenepura, Sri Lanka; b Department of Pharmacy and Pharmaceutical Sciences, University of Sri Jayewardenepura, Sri Lanka; c Department of Chemistry Louisiana State University,Baton Rouge LA 70803 USA; Katholieke Universiteit Leuven, Belgium

**Keywords:** rhenium complexes, di­ethyl­enetri­amine, biphen­yl, sulfonamide, crystal structure

## Abstract

The title compound, [Re(C_17_H_22_N_3_O_2_S)(CO)_3_] is a net neutral *fac*-Re(I)(CO)_3_ complex of the 4-methyl­biphenyl sulfonamide derivatized di­ethyl­enetri­amine ligand. The NNN-donor monoanionic ligand coordinates with the Re core in tridentate fashion, establishing an inner coordination sphere resulting in a net neutral complex. The complex possesses pseudo-octa­hedral geometry where one face of the octa­hedron is occupied by three carbonyl ligands and the other faces are occupied by one *sp*^2^ nitro­gen atom of the sulfonamide group and two *sp*^3^ nitro­gen atoms of the dien backbone. The Re—N*sp*^2^ bond distance, 2.173 (4) Å, is shorter than the Re—N*sp*^3^ bond distances, 2.217 (5) and 2.228 (6) Å, and is similar to the range reported for typical Re—N*sp*^2^ bond lengths (2.14 to 2.18 Å).

## Abstract 2

The title complex possesses pseudo-octa­hedral geometry where one face of the octa­hedron is occupied by three carbonyl ligands and the other face is occupied by one *sp*^2^ nitro­gen atom of the sulfonamide group and two *sp*^3^ nitro­gen atoms of the dien backbone.

## 1. Chemical context

Organometallic compounds have garnered significant inter­est due to their notable properties in cell imaging and anti­cancer applications. Particularly, Re complexes are noted for their kinetic inertness and large Stokes shift (Stephenson *et al.*, 2004 ▸; Guo *et al.*, 1997 ▸). Research has shown that tridentate ligand systems are more robust and possess better pharmacokinetics than those bearing bidentate ligands, leading to reduced side effects (Schibli *et al.*, 2000 ▸). Our focus involves a sulfonamide ligand, which has a di­ethyl­enetri­amine (dien) backbone and 4-methyl­biphenyl (4-Mebip) as the pendant group. The N(SO_2_)(4-Mebip)dienH ligand, along with its bidentate Pt^II^ complex have both been reported to exhibit remarkable anti­cancer properties against non-small lung cancer (Kaluthanthiri *et al.*, 2023 ▸). Motivated by its potential as a cytotoxic drug lead, here we have focused on rhenium, in its lowest oxidation state because it exhibits less reactivity toward species in the cellular environment (Schibli & Schubiger, 2002 ▸). Given rhenium’s soft metal center characteristics, a preference for soft donors, particularly nitro­gen, is observed and tridentate metal complexes featuring nitro­gen donors are commonly employed (Christoforou *et al.*, 2007 ▸; Kaushalya *et al.*, 2022 ▸; Darshani *et al.*, 2020 ▸). In this study, the Re(CO)_3_[N(SO_2_)(4-Mebip)dien] complex was successfully synthesized and its mol­ecular structure was confirmed by single-crystal X-ray diffraction analysis and ^1^H NMR spectroscopy. Furthermore, comprehensive characterization was conducted using FTIR, UV–vis, and fluorescence spectroscopic tech­niques.
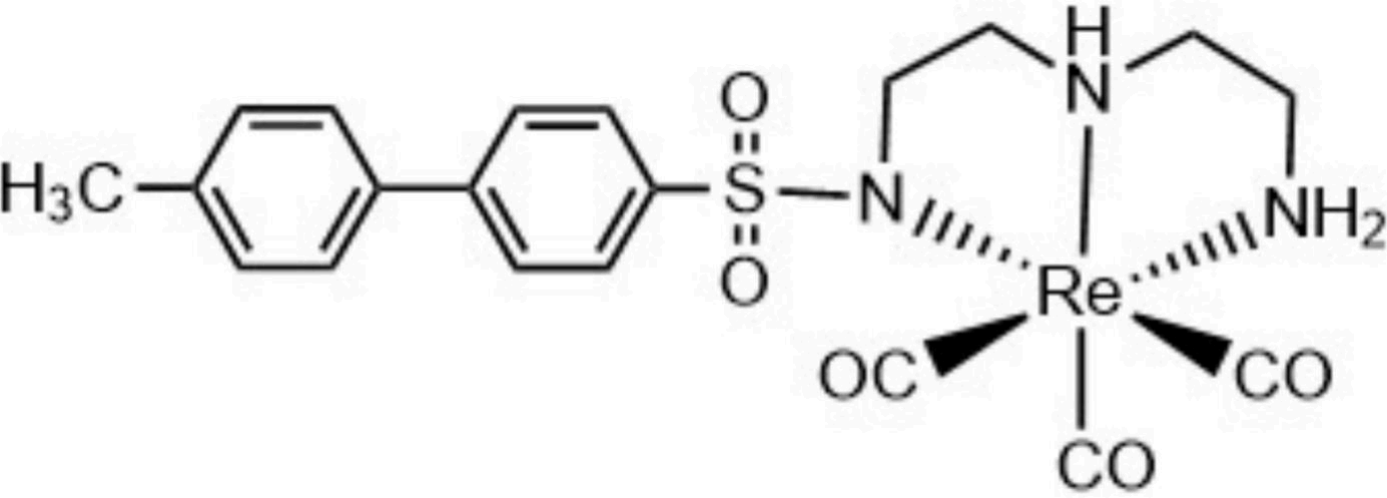


## 2. Structural commentary

**Figure 1:**
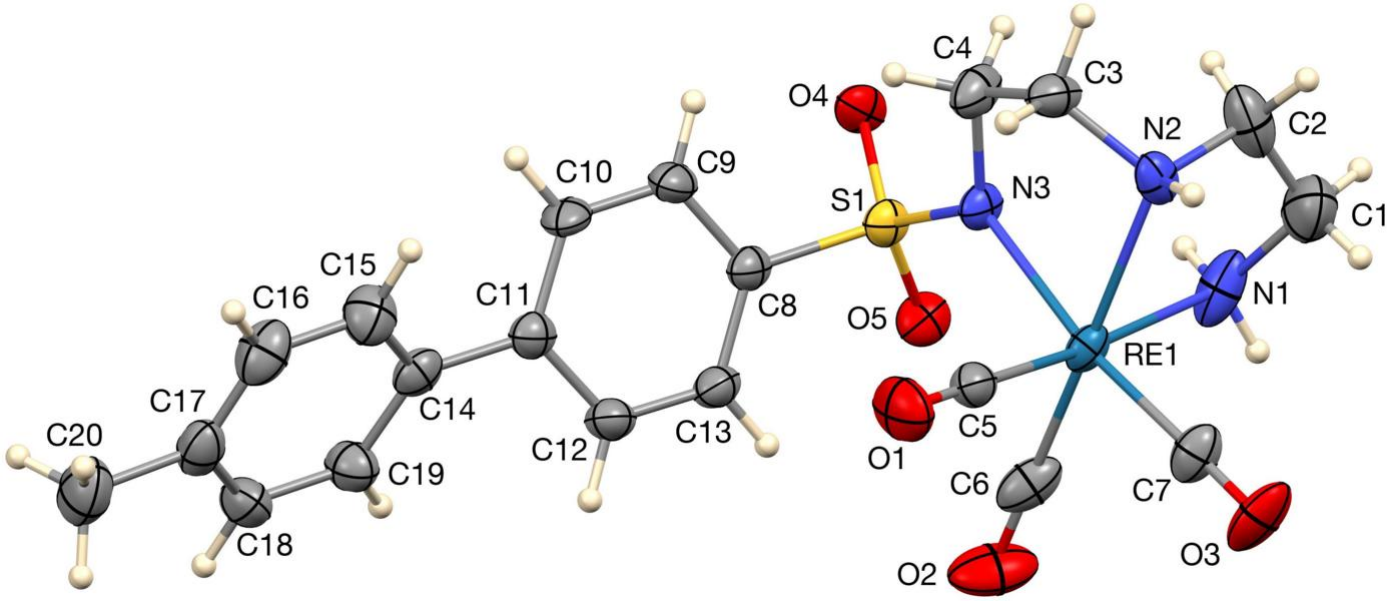
The asymmetric unit with 50% probability displacement ellipsoids and atom labels. H atoms are represented by spheres of arbitrary radius.

The Re(CO)_3_[N(SO_2_)(4-Mebip)dien] complex is shown in Fig. 1 ▸. The Re—C distances in Re—CO bonds are in the range of 1.895 (8)–1.914 (6) Å, which is consistent with related data reported (Christoforou *et al.*, 2007 ▸; Darshani *et al.*, 2020 ▸). The longest Re—C distance is *trans* to the *sp*^2^ nitro­gen atom N3. The C11—C14 bond distance between the two phenyl rings of the anionic ligand in the biphenyl group in Re(CO)_3_[N(SO_2_)(4-Mebip)dien] is 1.484 (8) Å. The biphenyl moiety is twisted out of planarity, the dihedral angle between the two planes being 36.5 (3)°. The average Re—N bond length in our Re complex is 2.206 Å, which is consistent with the distances found in related Re(CO)_3_ complexes containing a dien backbone (Christoforou *et al.*, 2007 ▸). The Re—N3 bond (*sp*^2^ nitro­gen) distance [2.173 (4) Å] in the complex is significantly shorter than the other Re—N (*sp*^3^ N) bonds [2.217 (5) and 2.228 (6) Å], which explains the anionic nature of the N3 amino nitro­gen. The S—N bond length for the deprotonated sulfonamido group is 1.579 (4) Å for the complex and is within the accepted range for S—N bonds available in deprotonated sulfonamides coordinated to Re, Cu and Zn metals (Christoforou *et al.*, 2007 ▸; Goodwin *et al.*, 2004 ▸; Congreve *et al.*, 2003 ▸).

## 3. Supra­molecular features

**Figure 2:**
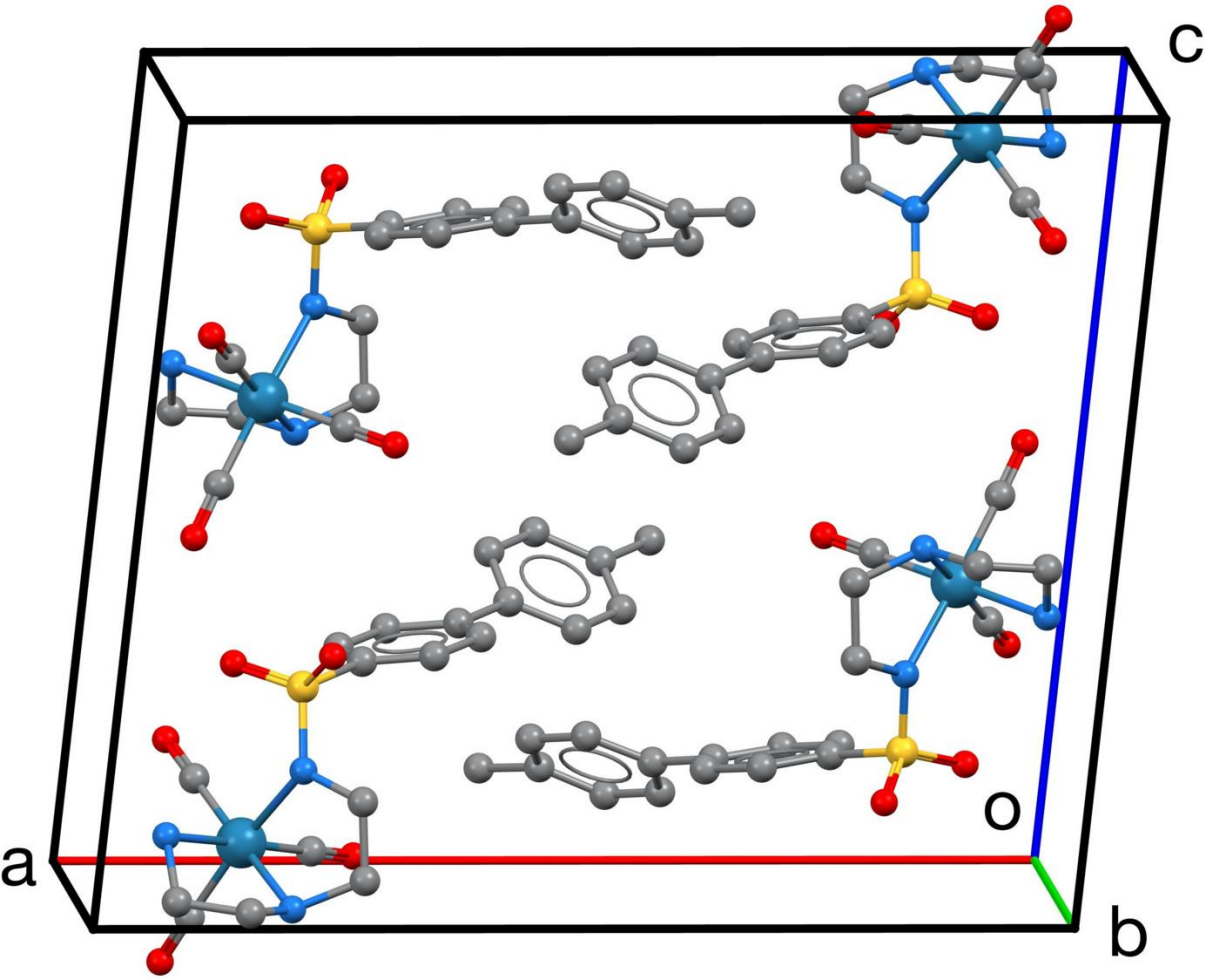
View of the unit cell. H atoms are not shown.

**Figure 3:**
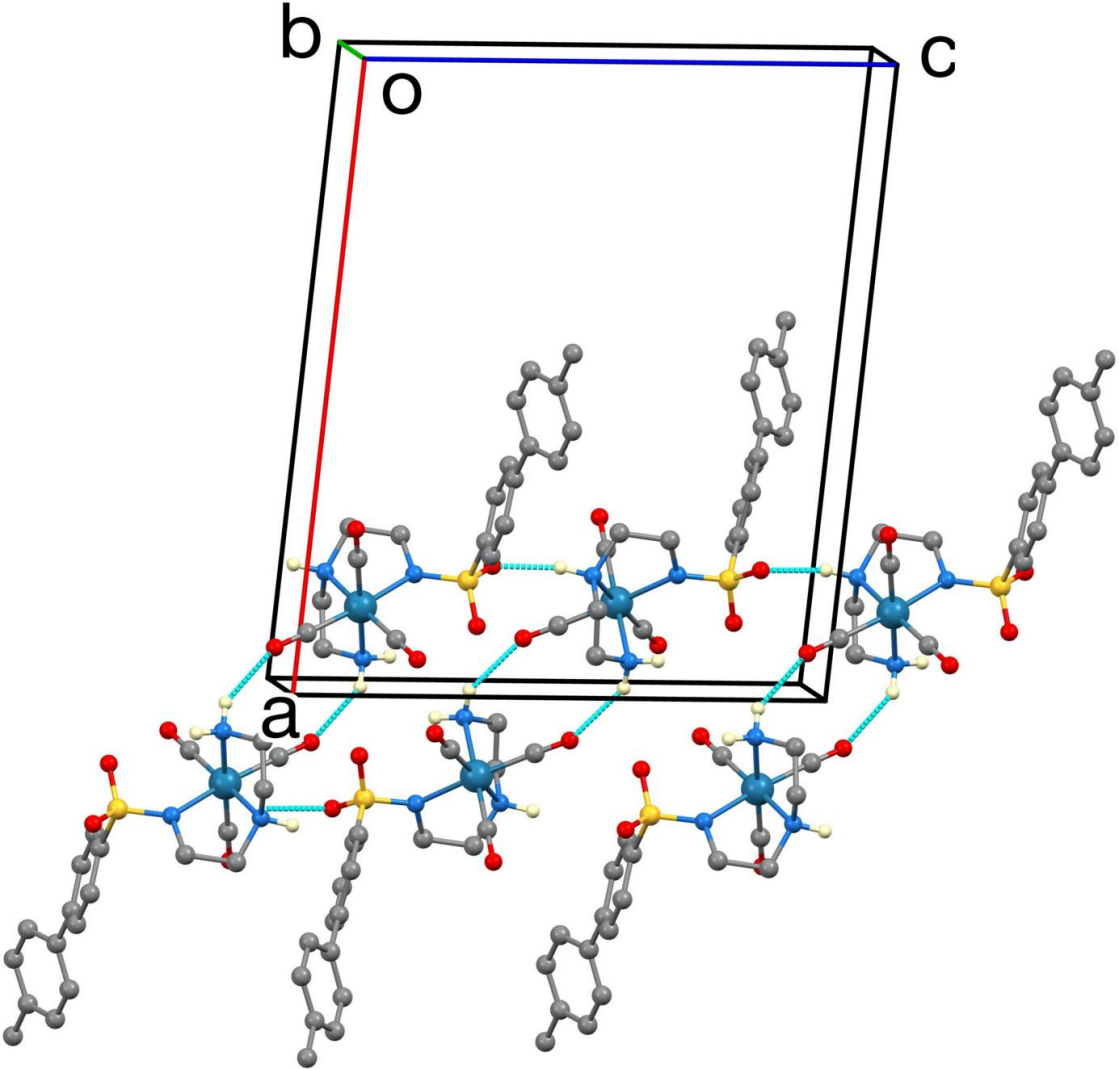
View of the hydrogen bonding, shown as blue dashed lines, and chain formation along the [001] direction. H atoms on C are omitted and the unit cell axes are shown.

The unit cell is shown in Fig. 2 ▸. The inter­molecular inter­actions are predominantly N—H⋯O hydrogen bonds as listed in Table 1 ▸ and shown in Fig. 3 ▸. The N1⋯O2 separations in these hydrogen bonds are in the range 2.941 (5)–3.053 (7) Å. The graph sets (Etter *et al.*, 1990 ▸) are centrosymmetric 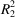
(10) rings and 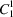
(6) chains, forming double-stranded chains in the [001] direction. One of the NH_2_ H atoms is not involved in the hydrogen bonding. Inter­leaved between the double-stranded hydrogen-bonded chains are hydro­phobic layers of stacked biphenyl moieties, as can be seen in Fig. 2 ▸. The closest distance [4.079 (4) Å] between centers of gravity (*Cg*) of these rings is between the phenyl ring C14–C19 carrying the methyl group and its centrosymmetric equivalent at 1 − *x*, 2 − *y*, 2 − *z*. There are no other *Cg*⋯*Cg* distances closer than 5.5 Å. The phenyl ring C8–C13 has no close inter­molecular contacts to other phenyl rings, but has a close contact to carbonyl C5—O1 at *x*, 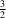 − *y*, −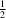 + *z*, with *Cg*⋯O1 3.758 (7) Å.

## 4. Database survey

A search of the Cambridge Structural Database (CSD, version 5.45, update of March 2024; Groom *et al.*, 2016 ▸) for the di­ethyl­enetri­amine SO_2_Re(CO)_3_ fragment yielded four hits, LIMDIV and LIMDOB (Christoforou *et al.*, 2007 ▸); SUNFUF and SUNGAM (Darshani *et al.*, 2020 ▸). These structures have been mentioned above. A similar search for [*N*-(2-amino­eth­yl)ethane-1,2-di­amine]Re(CO)_3_ salts yielded seven hits: BUPXAO, BUPXES, BUPYIX, and BUPYOD (Abhayawardhana *et al.*, 2020 ▸), IWENAZ (Mundwiler *et al.*, 2004 ▸), TIYVIH and TIYVON (Christoforou *et al.*, 2007 ▸). In these structures, the Re—N distances are in the range 2.203 (7)–2.244 (3) Å, with a mean value 2.219 Å for 21 individual measurements. The Re—C distances are in the range 1.878 (12)–1.956 (15) Å with a mean value 1.917 Å. There is no indication that the Re—N or Re—C distances to the central ligand atoms differ from those to the terminal atoms.

## 5. Synthesis, crystallization and spectroscopic data

**Figure 4:**
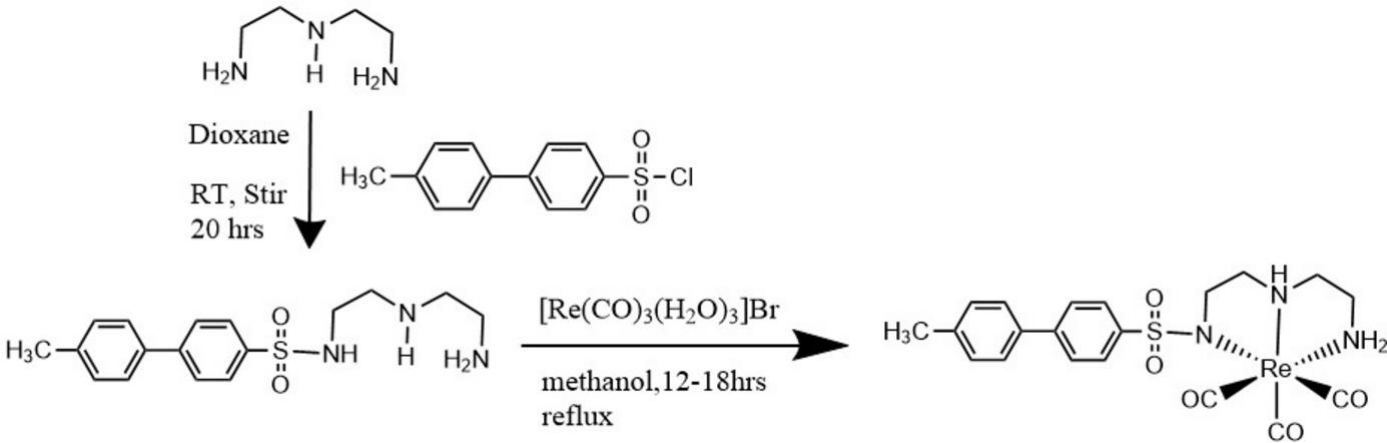
Synthetic route for preparation of N(SO_2_)(4-Mebip)dienH and Re(CO)_3_[N(SO_2_)(4-Mebip)dien].

**Figure 5:**
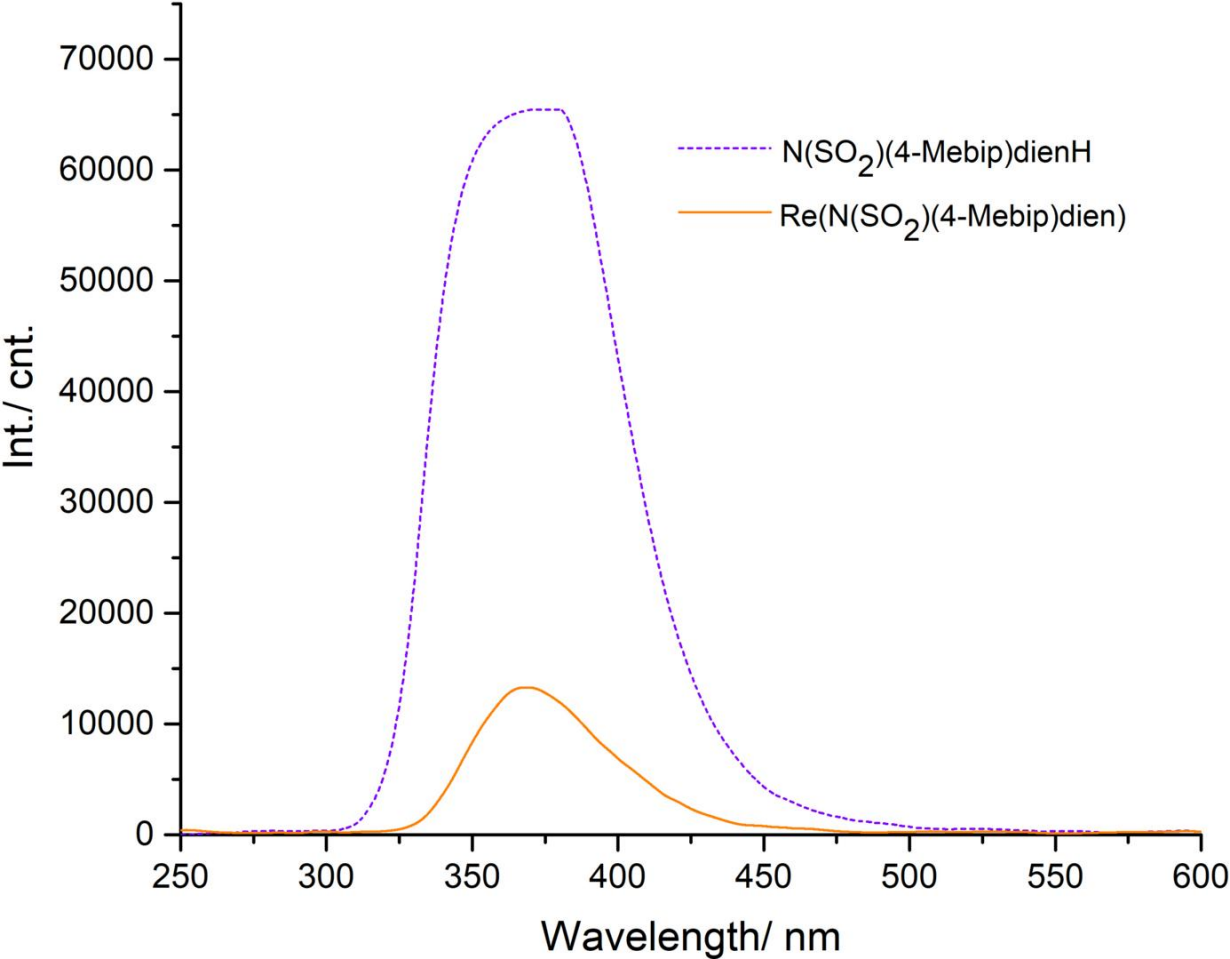
Fluorescence emission spectra of N(SO_2_)(4-Mebip)dienH and Re(CO)_3_[N(SO_2_)(4-Mebip)dien] in methanol at 298 K.

The ligand N(SO_2_)(4-Mebip)dienH was synthesized by following a reported procedure (Fig. 4 ▸; Kaluthanthiri *et al.*, 2023 ▸). A solution of the ligand N(SO_2_)(4-Mebip)dienH (0.0272 g, 0.0816 mmol) in 2 ml of methanol was added to a solution of [Re(CO)_3_(H_2_O)_3_]Br (0.033 g, 0.0816 mmol) in 3 ml of water. The solution was then adjusted to pH 7–8 with aqueous NaOH and refluxed for 16 h (Fig. 4 ▸). The complex formed was collected by filtration as a white powder (0.025 g, 51% yield). Crystals suitable for X-ray crystallography were grown by slow evaporation of an aceto­nitrile/ methanol solution. UV–vis (MeOH) [λ_max_ (nm)]: 203, 266; FT–IR (ATR) (cm^−1^): 969 ([(S—N)], 1342, 1128 [ν(S=O)], 2008, 1865 [ν(CO)]. ^1^H NMR (400 MHz, DMSO-*d*_6_) δ(ppm): 7.81 (*m*, 2H, Ha/a′), 7.74 (*m*, 2H, Hb/b′), 7.62 (*m*, 2H, Hc/c′) 7.29 (*m*, 2H, Hd/d′), 6.69 (*b*, 1H, N2H), 5.15 (*m*, 1H, *endo* N1H), 3.47 (*m*, 1H, *exo* N1H), 3.34–3.38 (*m*, 1H, CH), 2.64–2.90 (*m*, 7H, CH_2_), 2.35 (*s*, 3H, CH_3_). Although the ligand shows excellent fluorescence properties even at low concentrations, its Re complex offers quenched fluorescence properties attributed to the direct binding of sulfonamide nitro­gen to Re center in the complex (Fig. 5 ▸). Furthermore, a slight blue shift (about 9 nm) was observed in the complex.

## 6. Refinement

Crystal data, data collection and structure refinement details are summarized in Table 2 ▸. All H atoms were located in difference maps and treated as riding in geometrically ideal­ized positions with C—H distances of 0.94 Å and with *U*_iso_(H) = 1.2*U*_eq_ for the attached C atom (0.97 Å and 1.5*U*_eq_ for the methyl group). The H atoms on nitro­gen had N—H distances of 0.89 Å for NH_2_ and 0.98 Å for NH, and *U*_iso_ values were assigned as 1.2*U*_eq_ for the N atom.

## Supplementary Material

Crystal structure: contains datablock(s) I, global. DOI: 10.1107/S2056989024005656/vm2303sup1.cif

Structure factors: contains datablock(s) I. DOI: 10.1107/S2056989024005656/vm2303Isup2.hkl

Supporting information file. DOI: 10.1107/S2056989024005656/vm2303Isup3.cdx

CCDC reference: 2362252

Additional supporting information:  crystallographic information; 3D view; checkCIF report

## Figures and Tables

**Table 1:** Hydrogen-bond geometry (Å, °)

*D*—H⋯*A*	*D*—H	H⋯*A*	*D*⋯*A*	*D*—H⋯*A*
N1—H11*N*⋯O3^i^	0.89	2.27	3.053 (7)	146
N1—H12*N*⋯O2^ii^	0.89	2.59	3.028 (7)	111
N2—H2*N*⋯O4^iii^	0.98	1.98	2.941 (5)	167

Symmetry codes: (i) 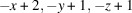; (ii) 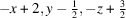; (iii) 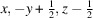.

**Table 2:** Experimental details

Crystal data	
Chemical formula	[Re(C_17_H_22_N_3_O_2_S)(CO)_3_]
*M*_r_	602.66
Crystal system, space group	Monoclinic, *P*2_1_/*c*
Temperature (K)	296
*a*, *b*, *c* (Å)	18.5651 (9), 7.6604 (4), 15.4897 (11)
β (°)	95.472 (2)
*V* (Å^3^)	2192.8 (2)
*Z*	4
Radiation type	Mo *K*α
μ (mm^−1^)	5.67
Crystal size (mm)	0.22 × 0.13 × 0.05

Data collection	
Diffractometer	Bruker Kappa APEXII CCD
Absorption correction	Multi-scan (*SADABS*; Krause *et al.*, 2015 ▸)
*T*_min_, *T*_max_	0.572, 0.765
No. of measured, independent and observed [*I* > 2σ(*I*)] reflections	119413, 4849, 3513
*R*_int_	0.102
(sin θ/λ)_max_ (Å^−1^)	0.643

Refinement	
*R*[*F*^2^ > 2σ(*F*^2^)], *wR*(*F*^2^), *S*	0.035, 0.071, 1.08
No. of reflections	4849
No. of parameters	273
H-atom treatment	H-atom parameters constrained
Δρ_max_, Δρ_min_ (e Å^−3^)	2.00, −1.80

Computer programs: *APEX3* and *SAINT* (Bruker, 2016 ▸), *SHELXT2018/2* (Sheldrick, 2015 ▸a), *SHELXL2018/1* (Sheldrick, 2015 ▸b), *Mercury* (Macrae *et al.*, 2020 ▸) and *publCIF* (Westrip, 2010 ▸).

## supplementary crystallographic information

### (N-{2-[(2-Aminoethyl)amino]ethyl}-4'-methyl-[1,1'-biphenyl]-4-sulfonamidato)tricarbonylrhenium(I) Crystal data

**Table:**

[Re(C_17_H_22_N_3_O_2_S)(CO)_3_]	*F*(000) = 1176
*M**_r_* = 602.66	*D*_x_ = 1.825 Mg m^−^^3^
Monoclinic, *P*2_1_/*c*	Mo *K*α radiation, λ = 0.71073 Å
*a* = 18.5651 (9) Å	Cell parameters from 2346 reflections
*b* = 7.6604 (4) Å	θ = 2.6–24.1°
*c* = 15.4897 (11) Å	µ = 5.67 mm^−^^1^
β = 95.472 (2)°	*T* = 296 K
*V* = 2192.8 (2) Å^3^	Needle, colorless
*Z* = 4	0.22 × 0.13 × 0.05 mm

### (N-{2-[(2-Aminoethyl)amino]ethyl}-4'-methyl-[1,1'-biphenyl]-4-sulfonamidato)tricarbonylrhenium(I) Data collection

**Table:**

Bruker Kappa APEXII CCD diffractometer	4849 independent reflections
Radiation source: fine-focus sealed tube	3513 reflections with *I* > 2σ(*I*)
TRIUMPH curved graphite monochromator	*R*_int_ = 0.102
φ and ω scans	θ_max_ = 27.2°, θ_min_ = 1.1°
Absorption correction: multi-scan (SADABS; Krause *et al.*, 2015)	*h* = −23→23
*T*_min_ = 0.572, *T*_max_ = 0.765	*k* = −9→9
119413 measured reflections	*l* = −19→19

### (N-{2-[(2-Aminoethyl)amino]ethyl}-4'-methyl-[1,1'-biphenyl]-4-sulfonamidato)tricarbonylrhenium(I) Refinement

**Table:**

Refinement on *F*^2^	Hydrogen site location: inferred from neighbouring sites
Least-squares matrix: full	H-atom parameters constrained
*R*[*F*^2^ > 2σ(*F*^2^)] = 0.035	*w* = 1/[σ^2^(*F*_o_^2^) + (0.0179*P*)^2^ + 6.5423*P*] where *P* = (*F*_o_^2^ + 2*F*_c_^2^)/3
*wR*(*F*^2^) = 0.071	(Δ/σ)_max_ = 0.001
*S* = 1.08	Δρ_max_ = 2.00 e Å^−^^3^
4849 reflections	Δρ_min_ = −1.80 e Å^−^^3^
273 parameters	Extinction correction: *SHELXL2017/1* (Sheldrick 2015b), Fc^*^=kFc[1+0.001xFc^2^λ^3^/sin(2θ)]^-1/4^
0 restraints	Extinction coefficient: 0.00017 (5)

### (N-{2-[(2-Aminoethyl)amino]ethyl}-4'-methyl-[1,1'-biphenyl]-4-sulfonamidato)tricarbonylrhenium(I) Special details

**Table:**

Geometry. All esds (except the esd in the dihedral angle between two l.s. planes) are estimated using the full covariance matrix. The cell esds are taken into account individually in the estimation of esds in distances, angles and torsion angles; correlations between esds in cell parameters are only used when they are defined by crystal symmetry. An approximate (isotropic) treatment of cell esds is used for estimating esds involving l.s. planes.

### (N-{2-[(2-Aminoethyl)amino]ethyl}-4'-methyl-[1,1'-biphenyl]-4-sulfonamidato)tricarbonylrhenium(I) Fractional atomic coordinates and isotropic or equivalent isotropic displacement parameters (Å^2^)

**Table:**

	*x*	*y*	*z*	*U*_iso_*/*U*_eq_	
Re1	0.86146 (2)	0.45261 (3)	0.61070 (2)	0.04344 (9)	
S1	0.82122 (8)	0.3572 (2)	0.81148 (8)	0.0456 (4)	
O1	0.7335 (3)	0.7018 (7)	0.5732 (4)	0.0875 (16)	
O2	0.9354 (3)	0.7562 (8)	0.7131 (3)	0.104 (2)	
O3	0.9230 (3)	0.6052 (8)	0.4508 (3)	0.0879 (17)	
O4	0.8016 (2)	0.2083 (5)	0.8619 (2)	0.0563 (11)	
O5	0.8906 (2)	0.4354 (6)	0.8338 (2)	0.0568 (11)	
N1	0.9482 (3)	0.2530 (9)	0.6360 (3)	0.0769 (19)	
H11N	0.991541	0.302049	0.634639	0.092*	
H12N	0.945892	0.205082	0.688023	0.092*	
N2	0.8135 (3)	0.2133 (6)	0.5486 (3)	0.0487 (12)	
H2N	0.801808	0.237533	0.486680	0.058*	
N3	0.8132 (2)	0.3099 (6)	0.7118 (2)	0.0440 (12)	
C1	0.9364 (5)	0.1159 (12)	0.5660 (6)	0.099 (3)	
H1C	0.949062	0.163356	0.511455	0.119*	
H1D	0.967551	0.016551	0.580794	0.119*	
C2	0.8629 (5)	0.0603 (10)	0.5568 (4)	0.080 (2)	
H2A	0.852992	−0.008484	0.606844	0.096*	
H2B	0.854413	−0.012739	0.505655	0.096*	
C3	0.7445 (4)	0.1752 (9)	0.5872 (4)	0.0598 (18)	
H3A	0.727485	0.059835	0.569374	0.072*	
H3B	0.707984	0.259311	0.565914	0.072*	
C4	0.7553 (4)	0.1838 (10)	0.6838 (4)	0.0655 (19)	
H4A	0.710554	0.219162	0.706318	0.079*	
H4B	0.768274	0.069138	0.706919	0.079*	
C5	0.7818 (4)	0.6059 (8)	0.5873 (4)	0.0525 (16)	
C6	0.9077 (4)	0.6386 (11)	0.6748 (4)	0.069 (2)	
C7	0.9009 (3)	0.5469 (10)	0.5110 (4)	0.0607 (17)	
C8	0.7558 (3)	0.5190 (8)	0.8291 (3)	0.0457 (14)	
C9	0.6854 (3)	0.4687 (8)	0.8401 (4)	0.0550 (16)	
H9	0.672952	0.351064	0.838917	0.066*	
C10	0.6339 (3)	0.5938 (8)	0.8529 (4)	0.0561 (17)	
H10	0.586941	0.558250	0.860192	0.067*	
C11	0.6498 (3)	0.7698 (8)	0.8553 (3)	0.0492 (15)	
C12	0.7211 (3)	0.8167 (8)	0.8449 (4)	0.0534 (16)	
H12	0.733858	0.934122	0.846868	0.064*	
C13	0.7730 (3)	0.6936 (8)	0.8319 (4)	0.0506 (15)	
H13	0.820003	0.728879	0.824931	0.061*	
C14	0.5948 (3)	0.9040 (8)	0.8704 (4)	0.0517 (16)	
C15	0.5233 (4)	0.8895 (10)	0.8371 (4)	0.070 (2)	
H15	0.508519	0.791583	0.804678	0.084*	
C16	0.4733 (4)	1.0176 (11)	0.8511 (5)	0.075 (2)	
H16	0.425596	1.003962	0.827930	0.090*	
C17	0.4927 (4)	1.1647 (10)	0.8984 (5)	0.0662 (19)	
C18	0.5630 (4)	1.1776 (9)	0.9336 (5)	0.0654 (18)	
H18	0.577106	1.273921	0.967606	0.079*	
C19	0.6130 (4)	1.0513 (9)	0.9197 (4)	0.0613 (16)	
H19	0.660456	1.064908	0.944103	0.074*	
C20	0.4383 (4)	1.3088 (11)	0.9120 (5)	0.092 (3)	
H20A	0.461891	1.420204	0.911398	0.138*	
H20B	0.419068	1.292329	0.966780	0.138*	
H20C	0.399557	1.304649	0.866244	0.138*	

### (N-{2-[(2-Aminoethyl)amino]ethyl}-4'-methyl-[1,1'-biphenyl]-4-sulfonamidato)tricarbonylrhenium(I) Atomic displacement parameters (Å^2^)

**Table:**

	*U*^11^	*U*^22^	*U*^33^	*U*^12^	*U*^13^	*U*^23^
Re1	0.04118 (13)	0.06022 (16)	0.02905 (11)	−0.00246 (13)	0.00405 (8)	0.00870 (12)
S1	0.0592 (9)	0.0527 (9)	0.0250 (6)	−0.0062 (8)	0.0056 (6)	0.0006 (6)
O1	0.070 (3)	0.073 (4)	0.118 (4)	0.023 (3)	0.002 (3)	−0.001 (3)
O2	0.113 (5)	0.112 (5)	0.079 (4)	−0.062 (4)	−0.029 (3)	0.013 (3)
O3	0.071 (3)	0.134 (5)	0.062 (3)	−0.002 (3)	0.023 (2)	0.050 (3)
O4	0.090 (3)	0.052 (3)	0.0283 (19)	−0.006 (2)	0.012 (2)	0.0072 (18)
O5	0.056 (2)	0.074 (3)	0.039 (2)	−0.009 (2)	−0.0056 (18)	−0.002 (2)
N1	0.044 (3)	0.138 (6)	0.049 (3)	0.022 (3)	0.008 (3)	0.029 (4)
N2	0.071 (3)	0.047 (3)	0.028 (2)	0.012 (3)	0.006 (2)	0.004 (2)
N3	0.055 (3)	0.055 (3)	0.023 (2)	−0.009 (2)	0.0078 (19)	−0.002 (2)
C1	0.085 (6)	0.093 (7)	0.126 (8)	0.017 (5)	0.041 (6)	0.006 (6)
C2	0.112 (7)	0.076 (5)	0.052 (4)	0.042 (5)	0.008 (4)	−0.002 (4)
C3	0.080 (5)	0.056 (4)	0.044 (3)	−0.025 (4)	0.010 (3)	−0.003 (3)
C4	0.070 (4)	0.087 (5)	0.041 (3)	−0.027 (4)	0.016 (3)	−0.015 (3)
C5	0.059 (4)	0.049 (4)	0.051 (4)	−0.009 (3)	0.013 (3)	−0.004 (3)
C6	0.056 (4)	0.100 (6)	0.050 (4)	−0.017 (4)	−0.004 (3)	0.027 (4)
C7	0.053 (4)	0.084 (5)	0.045 (3)	0.005 (4)	0.006 (3)	0.016 (4)
C8	0.057 (4)	0.050 (4)	0.031 (3)	−0.012 (3)	0.010 (2)	−0.005 (2)
C9	0.070 (4)	0.043 (3)	0.053 (3)	−0.019 (4)	0.016 (3)	−0.011 (3)
C10	0.050 (4)	0.055 (4)	0.065 (4)	−0.028 (3)	0.014 (3)	−0.015 (3)
C11	0.060 (4)	0.052 (4)	0.036 (3)	−0.007 (3)	0.008 (3)	−0.001 (3)
C12	0.064 (4)	0.044 (4)	0.053 (4)	−0.012 (3)	0.011 (3)	0.001 (3)
C13	0.053 (4)	0.054 (4)	0.047 (3)	−0.015 (3)	0.012 (3)	−0.001 (3)
C14	0.053 (4)	0.062 (4)	0.040 (3)	−0.006 (3)	0.008 (3)	0.009 (3)
C15	0.065 (5)	0.087 (6)	0.057 (4)	−0.003 (4)	0.001 (3)	−0.011 (4)
C16	0.053 (4)	0.109 (7)	0.063 (4)	0.001 (4)	0.002 (3)	0.005 (4)
C17	0.069 (5)	0.069 (5)	0.065 (4)	0.011 (4)	0.025 (4)	0.016 (4)
C18	0.068 (5)	0.046 (4)	0.083 (5)	0.002 (4)	0.013 (4)	−0.002 (4)
C19	0.056 (4)	0.055 (4)	0.072 (4)	−0.001 (4)	0.002 (3)	−0.003 (4)
C20	0.075 (5)	0.100 (7)	0.106 (6)	0.019 (5)	0.030 (5)	0.012 (5)

### (N-{2-[(2-Aminoethyl)amino]ethyl}-4'-methyl-[1,1'-biphenyl]-4-sulfonamidato)tricarbonylrhenium(I) Geometric parameters (Å, º)

**Table:**

Re1—C6	1.895 (8)	C4—H4A	0.9700
Re1—C5	1.896 (7)	C4—H4B	0.9700
Re1—C7	1.914 (6)	C8—C13	1.375 (8)
Re1—N3	2.173 (4)	C8—C9	1.389 (8)
Re1—N2	2.217 (5)	C9—C10	1.381 (9)
Re1—N1	2.228 (6)	C9—H9	0.9300
S1—O5	1.433 (4)	C10—C11	1.381 (8)
S1—O4	1.448 (4)	C10—H10	0.9300
S1—N3	1.579 (4)	C11—C12	1.394 (8)
S1—C8	1.775 (6)	C11—C14	1.484 (8)
O1—C5	1.163 (7)	C12—C13	1.376 (8)
O2—C6	1.170 (9)	C12—H12	0.9300
O3—C7	1.143 (7)	C13—H13	0.9300
N1—C1	1.510 (10)	C14—C15	1.381 (9)
N1—H11N	0.8900	C14—C19	1.387 (9)
N1—H12N	0.8900	C15—C16	1.381 (10)
N2—C2	1.486 (8)	C15—H15	0.9300
N2—C3	1.493 (7)	C16—C17	1.372 (10)
N2—H2N	0.9800	C16—H16	0.9300
N3—C4	1.479 (7)	C17—C18	1.370 (9)
C1—C2	1.423 (10)	C17—C20	1.524 (9)
C1—H1C	0.9700	C18—C19	1.372 (9)
C1—H1D	0.9700	C18—H18	0.9300
C2—H2A	0.9700	C19—H19	0.9300
C2—H2B	0.9700	C20—H20A	0.9600
C3—C4	1.493 (8)	C20—H20B	0.9600
C3—H3A	0.9700	C20—H20C	0.9600
C3—H3B	0.9700		
			
C6—Re1—C5	86.6 (3)	H3A—C3—H3B	108.1
C6—Re1—C7	87.1 (3)	N3—C4—C3	110.3 (5)
C5—Re1—C7	87.9 (3)	N3—C4—H4A	109.6
C6—Re1—N3	101.4 (2)	C3—C4—H4A	109.6
C5—Re1—N3	94.7 (2)	N3—C4—H4B	109.6
C7—Re1—N3	171.2 (2)	C3—C4—H4B	109.6
C6—Re1—N2	172.9 (2)	H4A—C4—H4B	108.1
C5—Re1—N2	99.0 (2)	O1—C5—Re1	179.1 (6)
C7—Re1—N2	97.5 (2)	O2—C6—Re1	178.4 (7)
N3—Re1—N2	73.78 (16)	O3—C7—Re1	178.4 (6)
C6—Re1—N1	98.1 (3)	C13—C8—C9	119.0 (6)
C5—Re1—N1	174.9 (3)	C13—C8—S1	121.6 (5)
C7—Re1—N1	94.3 (2)	C9—C8—S1	119.4 (5)
N3—Re1—N1	82.45 (18)	C10—C9—C8	119.9 (6)
N2—Re1—N1	76.3 (2)	C10—C9—H9	120.1
O5—S1—O4	117.7 (3)	C8—C9—H9	120.1
O5—S1—N3	109.4 (2)	C11—C10—C9	122.1 (6)
O4—S1—N3	110.0 (2)	C11—C10—H10	118.9
O5—S1—C8	106.5 (3)	C9—C10—H10	118.9
O4—S1—C8	104.8 (3)	C10—C11—C12	116.9 (6)
N3—S1—C8	108.0 (3)	C10—C11—C14	122.1 (6)
C1—N1—Re1	107.3 (4)	C12—C11—C14	121.0 (6)
C1—N1—H11N	110.2	C13—C12—C11	121.7 (6)
Re1—N1—H11N	110.2	C13—C12—H12	119.2
C1—N1—H12N	110.2	C11—C12—H12	119.2
Re1—N1—H12N	110.2	C8—C13—C12	120.5 (6)
H11N—N1—H12N	108.5	C8—C13—H13	119.8
C2—N2—C3	111.0 (5)	C12—C13—H13	119.8
C2—N2—Re1	113.3 (4)	C15—C14—C19	116.5 (6)
C3—N2—Re1	108.2 (3)	C15—C14—C11	122.5 (6)
C2—N2—H2N	108.1	C19—C14—C11	120.9 (6)
C3—N2—H2N	108.1	C14—C15—C16	121.4 (7)
Re1—N2—H2N	108.1	C14—C15—H15	119.3
C4—N3—S1	115.8 (3)	C16—C15—H15	119.3
C4—N3—Re1	117.1 (3)	C17—C16—C15	121.4 (7)
S1—N3—Re1	125.6 (3)	C17—C16—H16	119.3
C2—C1—N1	110.7 (6)	C15—C16—H16	119.3
C2—C1—H1C	109.5	C18—C17—C16	117.5 (7)
N1—C1—H1C	109.5	C18—C17—C20	120.8 (7)
C2—C1—H1D	109.5	C16—C17—C20	121.7 (7)
N1—C1—H1D	109.5	C17—C18—C19	121.4 (7)
H1C—C1—H1D	108.1	C17—C18—H18	119.3
C1—C2—N2	110.5 (7)	C19—C18—H18	119.3
C1—C2—H2A	109.5	C18—C19—C14	121.7 (6)
N2—C2—H2A	109.5	C18—C19—H19	119.1
C1—C2—H2B	109.5	C14—C19—H19	119.1
N2—C2—H2B	109.5	C17—C20—H20A	109.5
H2A—C2—H2B	108.1	C17—C20—H20B	109.5
N2—C3—C4	110.8 (5)	H20A—C20—H20B	109.5
N2—C3—H3A	109.5	C17—C20—H20C	109.5
C4—C3—H3A	109.5	H20A—C20—H20C	109.5
N2—C3—H3B	109.5	H20B—C20—H20C	109.5
C4—C3—H3B	109.5		
			
O5—S1—N3—C4	162.7 (5)	C8—C9—C10—C11	0.0 (9)
O4—S1—N3—C4	32.0 (5)	C9—C10—C11—C12	−0.6 (9)
C8—S1—N3—C4	−81.8 (5)	C9—C10—C11—C14	−179.1 (6)
O5—S1—N3—Re1	−31.7 (4)	C10—C11—C12—C13	0.7 (9)
O4—S1—N3—Re1	−162.5 (3)	C14—C11—C12—C13	179.3 (5)
C8—S1—N3—Re1	83.7 (4)	C9—C8—C13—C12	−0.4 (8)
Re1—N1—C1—C2	−48.9 (8)	S1—C8—C13—C12	179.4 (4)
N1—C1—C2—N2	49.9 (9)	C11—C12—C13—C8	−0.3 (9)
C3—N2—C2—C1	−148.2 (6)	C10—C11—C14—C15	−37.0 (9)
Re1—N2—C2—C1	−26.3 (7)	C12—C11—C14—C15	144.5 (6)
C2—N2—C3—C4	76.7 (7)	C10—C11—C14—C19	142.6 (6)
Re1—N2—C3—C4	−48.2 (6)	C12—C11—C14—C19	−35.9 (8)
S1—N3—C4—C3	169.7 (5)	C19—C14—C15—C16	1.4 (10)
Re1—N3—C4—C3	2.9 (7)	C11—C14—C15—C16	−179.1 (6)
N2—C3—C4—N3	29.9 (8)	C14—C15—C16—C17	0.1 (11)
O5—S1—C8—C13	20.8 (5)	C15—C16—C17—C18	−1.9 (10)
O4—S1—C8—C13	146.3 (5)	C15—C16—C17—C20	178.3 (7)
N3—S1—C8—C13	−96.5 (5)	C16—C17—C18—C19	2.2 (10)
O5—S1—C8—C9	−159.4 (4)	C20—C17—C18—C19	−178.0 (6)
O4—S1—C8—C9	−33.9 (5)	C17—C18—C19—C14	−0.7 (11)
N3—S1—C8—C9	83.3 (5)	C15—C14—C19—C18	−1.1 (10)
C13—C8—C9—C10	0.5 (9)	C11—C14—C19—C18	179.3 (6)
S1—C8—C9—C10	−179.3 (5)		

### (N-{2-[(2-Aminoethyl)amino]ethyl}-4'-methyl-[1,1'-biphenyl]-4-sulfonamidato)tricarbonylrhenium(I) Hydrogen-bond geometry (Å, º)

**Table:**

*D*—H···*A*	*D*—H	H···*A*	*D*···*A*	*D*—H···*A*
N1—H11*N*···O3^i^	0.89	2.27	3.053 (7)	146
N1—H12*N*···O2^ii^	0.89	2.59	3.028 (7)	111
N2—H2*N*···O4^iii^	0.98	1.98	2.941 (5)	167

Symmetry codes: (i) −*x*+2, −*y*+1, −*z*+1; (ii) −*x*+2, *y*−1/2, −*z*+3/2; (iii) *x*, −*y*+1/2, *z*−1/2.

## References

[bb1] Abhayawardhana, P., Marzilli, L. G. & Fronczek, F. R. (2020). *CSD Communications* (refcodes BUPXAO, BUPXES, BUPYIX, and BUPYOD). CCDC, Cambridge, England.

[bb2] Bruker (2016). *APEX3* and *SAINT*. Bruker AXS Inc., Madison, Wisconsin, USA.

[bb3] Christoforou, A. M., Marzilli, P. A., Fronczek, F. R. & Marzilli, L. G. (2007). *Inorg. Chem.* **46**, 11173–11182. 10.1021/ic701576u 18044880

[bb4] Congreve, A., Kataky, R., Knell, M., Parker, D., Puschmann, H., Senanayake, K. & Wylie, L. (2003). *New J. Chem.* **27**, 98–106.

[bb5] Darshani, T., Fronczek, F. R., Priyadarshani, V. V., Samarakoon, S. R., Perera, I. C. & Perera, T. (2020). *Polyhedron*, **187**, 114652.

[bb6] Etter, M. C., MacDonald, J. C. & Bernstein, J. (1990). *Acta Cryst.* B**46**, 256–262. 10.1107/s0108768189012929 2344397

[bb7] Goodwin, J. M., Olmstead, M. M. & Patten, T. (2004). *J. Am. Chem. Soc.* **126**, 14352–14353. 10.1021/ja045003g 15521744

[bb8] Groom, C. R., Bruno, I. J., Lightfoot, M. P. & Ward, S. C. (2016). *Acta Cryst.* B**72**, 171–179. 10.1107/S2052520616003954 PMC4822653 27048719

[bb9] Guo, X.-Q., Castellano, F. N., Li, L., Szmacinski, H., Lakowicz, J. R. & Sipior, J. (1997). *Anal. Biochem.* **254**, 179–186. 10.1006/abio.1997.2413 PMC6915065 9417774

[bb10] Kaluthanthiri, D., Rajagopalan, U., Samarakoon, S., Weerasinghe, L., Perera, I. C. & Perera, T. (2023). *Curr. Sci.* **26**, No. 02, 78–98.

[bb11] Kaushalya, C., Darshani, T., Samarakoon, S. R., Fronczek, F. R., Perera, I. C. & Perera, T. (2022). *J. Sci.* **25**, 103–121.

[bb12] Krause, L., Herbst-Irmer, R., Sheldrick, G. M. & Stalke, D. (2015). *J. Appl. Cryst.* **48**, 3–10. 10.1107/S1600576714022985 PMC4453166 26089746

[bb13] Macrae, C. F., Sovago, I., Cottrell, S. J., Galek, P. T. A., McCabe, P., Pidcock, E., Platings, M., Shields, G. P., Stevens, J. S., Towler, M. & Wood, P. A. (2020). *J. Appl. Cryst.* **53**, 226–235. 10.1107/S1600576719014092 PMC6998782 32047413

[bb14] Mundwiler, S., Candreia, L., Häfliger, P., Ortner, K. & Alberto, R. (2004). *Bioconjugate Chem.* **15**, 195–202. 10.1021/bc034171f 14733600

[bb15] Schibli, R., La Bella, R., Alberto, R., Garcia-Garayoa, E., Ortner, K., Abram, U. & Schubiger, P. A. (2000). *Bioconjugate Chem.* **11**, 345–351. 10.1021/bc990127h 10821650

[bb16] Schibli, R. & Schubiger, A. P. (2002). *Eur. J. Nucl. Med. Mol. Imaging*, **29**, 1529–1542. 10.1007/s00259-002-0900-8 12397472

[bb17] Sheldrick, G. M. (2015). *Acta Cryst.* C**71**, 3–8.

[bb18] Stephenson, K. A., Banerjee, S. R., Besanger, T., Sogbein, O. O., Levadala, M. K., McFarlane, N., Lemon, J. A., Boreham, D. R., Maresca, K. P., Brennan, J. D., Babich, J. W., Zubieta, J. & Valliant, J. F. (2004). *J. Am. Chem. Soc.* **126**, 8598–8599. 10.1021/ja047751b 15250681

[bb19] Westrip, S. P. (2010). *J. Appl. Cryst.* **43**, 920–925.

